# MK-STYX Alters the Morphology of Primary Neurons, and Outgrowths in MK-STYX Overexpressing PC-12 Cells Develop a Neuronal Phenotype

**DOI:** 10.3389/fmolb.2017.00076

**Published:** 2017-11-16

**Authors:** Dallas A. Banks, Arya Dahal, Alexander G. McFarland, Brittany M. Flowers, Christina A. Stephens, Benjamin Swack, Ayele Gugssa, Winston A. Anderson, Shantá D. Hinton

**Affiliations:** ^1^Department of Biology, Integrated Science Center, College of William and Mary, Williamsburg, VA, United States; ^2^National Cancer Institute, National Institutes of Health, Bethesda, MD, United States; ^3^Department of Chemistry, Integrated Science Center, College of William and Mary, Williamsburg, VA, United States; ^4^Department of Biology, Howard University, Washington, DC, United States

**Keywords:** pseudophosphatase, neuronal differentiation, neurite, synapses, Tau-1, MAP-2

## Abstract

We previously reported that the pseudophosphatase MK-STYX (mitogen activated kinase phosphoserine/threonine/tyrosine binding protein) dramatically increases the number of what appeared to be primary neurites in rat pheochromocytoma (PC-12) cells; however, the question remained whether these MK-STYX-induced outgrowths were *bona fide* neurites, and formed synapses. Here, we report that microtubules and microfilaments, components of the cytoskeleton that are involved in the formation of neurites, are present in MK-STYX-induced outgrowths. In addition, in response to nerve growth factor (NGF), MK-STYX-expressing cells produced more growth cones than non-MK-STYX-expressing cells, further supporting a model in which MK-STYX has a role in actin signaling. Furthermore, immunoblot analysis demonstrates that MK-STYX modulates actin expression. Transmission electron microscopy confirmed that MK-STYX-induced neurites form synapses. To determine whether these MK-STYX-induced neurites have pre-synaptic or post-synaptic properties, we used classical markers for axons and dendrites, Tau-1 and MAP2 (microtubule associated protein 2), respectively. MK-STYX induced neurites were dopaminergic and expression of both Tau-1 and MAP2 suggests that they have both axonal and dendritic properties. Further studies in rat hippocampal primary neurons demonstrated that MK-STYX altered their morphology. A significant number of primary neurons in the presence of MK-STYX had more than the normal number of primary neurites. Our data illustrate the novel findings that MK-STYX induces outgrowths in PC-12 cells that fit the criteria for neurites, have a greater number of growth cones, form synapses, and have pre-synaptic and post-synaptic properties. It also highlights that the pseudophosphatase MK-STYX significantly alters the morphology of primary neurons.

## Key findings

1. MK-STYX significantly increases the number of primary neurites in PC-12 cells.
2. MK-STYX-induced neurites exhibit growth cones and small actin protrusions.
3. MK-STYX modulates actin expression.
4. MK-STYX induced neurites form synapses.
5. MK-STYX-induced neurites form pre-synaptic and post-synaptic processes.
6. MK-STYX alters the morphology of hippocampal primary neurons.
7. MK-STYX increases the number of neurites in primary hippocampal neurons.

## Introduction

Phosphorylation cascades are critical regulators of various signaling processes, such as metabolism, proliferation, and differentiation. Kinases and phosphatases orchestrate the post-translational modification of proteins required to accomplish these cellular processes (Hornberg et al., 2005). Controlling such signaling pathways was initially thought to be a simple linear arrangement of the addition or removal of phosphate groups to or from proteins. However, it is now clear that phosphorylation cascades are complex networks encompassing multiple signaling molecules that function to elicit a cellular behavior, such as cytoskeletal rearrangement. Furthermore, it is becoming increasingly apparent that pseudoenzymes of kinases and phosphatases are integral components of signaling pathways as well (Reiterer et al., 2014), providing even more complexity to these cascades. Protein tyrosine phosphatase pseudoenzymes, referred to as pseudophosphatases, have been implicated in a number of cellular pathways, such as spermatogenesis, tumor suppression, oocyte maturation, and ubiquitination (Wishart and Dixon, 2002; Siligan et al., 2005; Parry et al., 2009; Reiterer et al., 2017).

Among these pseudophosphatases is a catalytically inactive member of the MKP (MAPK phosphatase) family, MK-STYX (Hinton et al., 2010). MK-STYX does not possess the active site signature motif **HC**(X_5_)**R** that is essential for phosphatase activity; it has the sequence I***FS***TQGIS**R**S, which renders it catalytically inactive (Wishart and Dixon, 1998; Tonks, 2006; Hinton et al., 2010). Despite being catalytically inactive, MK-STYX plays important roles in the cell. For example, MK-STYX binds G3BP-1 (Ras-GTPase activating protein SH3 domain binding protein-1) and inhibits stress granule formation (Hinton et al., 2010; Barr et al., 2013). It is also a master regulator of apoptotic potential of mitochondria by negatively regulating PTPM1 (PTP localized to the mitochondrion) (Niemi et al., 2011, 2014). Recently, we reported that it induces neurite-like outgrowths in rat adrenal pheochromocytoma (PC-12) cells through the RhoA signaling pathway (Flowers et al., 2014). MK-STYX over-expression decreased RhoA activation, while RhoA activation increased when MK-STYX was down-regulated. In addition, it affected the RhoA downstream target cofilin. Cofilin dephosphorylation is required for the induction of neurites (Zhang et al., 2006). MK-STYX decreased cofilin phosphorylation in the absence of NGF (Flowers et al., 2014), but increased cofilin phosphorylation upon NGF stimulation (Flowers et al., 2014). In addition to inducing outgrowths, when PC-12 cells expressing MK-STYX were stimulated with NGF, longer neurites formed and more branching occurred (Flowers et al., 2014; Dahal and Hinton, 2017), pointing toward the intriguing possibility that these cells were developing a neuronal phenotype.

PC-12 cells have been extensively used as a model system for neuronal differentiation because they differentiate in response to neurotrophins (Greene and Tischler, 1976; Keegan and Halegoua, 1993; Greene and Kaplan, 1995; Dixon et al., 2006). This cell line was derived from rat embryonic neural crest, which has a mixture of eosinophilic and neuroblastic cells that allow PC-12 cells to differentiate into neuron-like cells (Greene and Tischler, 1976). Thus, it was imperative to determine whether the neurite-like outgrowths originating from MK-STYX-expressing PC-12 cells that we observed previously (Flowers et al., 2014; Dahal and Hinton, 2017) formed connections and had pre-synaptic and post-synaptic properties. PC-12 cells release neurotransmitters, such as dopamine and acetylcholine (Schubert and Klier, 1977) and form typical synapses with neurons in primary culture (Schubert et al., 1977; Zhou et al., 2006). Furthermore, PC-12 cell neurites connect to each other, indicative of synapses, when stimulated by factors, such as NGF (Jeon et al., 2010). Moreover, neurites formed in PC-12 cells increase in response to various signaling pathways that promote neuronal differentiation (Sarma et al., 2015). For example, the heterotrimeric G protein, G_αs_ (guanine nucleotide binding protein alpha subunit) activates microtubules to increase neuronal growth in both primary hippocampal neurons and PC-12 cells (Sarma et al., 2015).

In this study we focused on whether MK-STYX-induced outgrowths in PC-12 cells develop into *bona fide* neurites. Data presented here show that MK-STYX significantly increased the number of primary neurites, and microfilament staining showed that more growth cones, which guide neurites to their synaptic partners (Kolodkin and Tessier-Lavigne, 2011; Santiago-Medina et al., 2015), formed in cells expressing MK-STYX. MK-STYX –induced neurite connections formed synapse-like structures. In addition, we identified Tau1 and MAP2, markers of axons and dendrites, respectively, in MK-STYX-induced neurites, showing that they had axonal and dendritic processes. Cells that branched connected at a Tau-1 and MAP2 neurite, suggesting that a pre-synaptic process was interacting with a post-synaptic process. In addition, dopamine, which is a neurotransmitter released in PC-12 cells (Schubert and Klier, 1977) was released in the presence of MK-STYX. The effects of MK-STYX in altering cell morphology, and initiating neurite formation were also observed in hippocampal primary neurons; MK-STYX significantly increased neurites in primary neurons. These findings provide further insight into the role of MK-STYX in neuronal development and a foundation for further investigation of the mechanism by which MK-STYX induces neurites.

## Materials and methods

### Cell culture and transient transfection

PC-12 (rat adrenal pheochromocytoma) cells (ATCC) were maintained at 37°C, 5% CO_2_ in Roswell Memorial Institute (RPMI) medium (Gibco, Invitrogen) supplemented with 10% horse serum (Invitrogen) and 5% fetal bovine serum (FBS) (Invitrogen). Using Lipofectamine 2000 (Invitrogen), cells were transfected with expression plasmids pMT2, pEGFP, mCherry, pMT2-FLAG-MK-STYX-FLAG, GFP-MK-STYX, or mCherry-MK-STYX. Cells were either not stimulated or stimulated with nerve growth factor (NGF), and analyzed with fluorescence microscopy.

### Live cell imaging and scoring cells

Cells were seeded at 1.5 × 10^5^ cells in a 60 mm plate (Fisher) and transfected 16 or 24 h post seeding. Live cell imaging of EGFP-expressing cells was conducted with phase contrast and fluorescence microscopy using a Nikon ECLIPSE Ti inverted fluorescence microscope. Cells were observed over a 3-day period, and scored day 3 post-treatment for “neurites,” defined as neurite-like outgrowths ≥ 20 μm in length. Cells were scored at least by day 3, when the outgrowths were clearly visible. Neurite outgrowth length was measured with NIS-Elements Basic Research software (version 3.10, Nikon). At least three replicate transfections were performed and at least 100 cells were scored per replicate. Samples were scored blind with regard to treatment and were scored independently by at least two different individuals. Cells were initially scored into two categories: no neurites and neurites. However, for the primary neurite distribution, cells were categorized as 1, 2, 3, 4, or ≥ 5 neurites (protrusions from the cell body ≥ 20 μm in length).

### NGF stimulation

Twenty-four hour post-transfection, PC-12 cells were serum-starved in Dulbecco's Modified Eagle Medium (DMEM) supplemented with 0.1% FBS for 8–12 h and then stimulated with 100 ng/ml of NGF (Prospec) (Li et al., 1998), followed by live cell imaging or immediate fixation for immunofluorescence studies.

### Immunoblotting

PC-12 cells were transfected with pMT2 or pMT2-FLAG-MK-STYX-FLAG expression plasmids, stimulated with NGF 2-days post-transfection, lysed 3 days post NGF stimulation, and analyzed by western blotting. Cells were harvested in lysis buffer [50 mM HEPES, pH 7.2, 150 mM NaCl, 10% glycerol, 10 mM NaF, 1% Nonidet P-40 alternative (Calbiochem), and protease inhibitor cocktail tablets (Roche)]. Lysates were centrifuged at 14,000 × g for 10 min, and the supernatant protein concentration was determined by NanoDrop quantification. Lysates were resolved by 10% SDS-PAGE and transferred to PVDF membrane by iBlot (Invitrogen) for immunoblot analysis with anti-actin (C4) (Santa Cruz), anti-FLAG (Sigma), or anti-β tubulin (Pierce) antibodies, followed by chemiluminescent detection. When warranted, blots were stripped (200 mM glycine, 3.5 mM SDS, 1% Tween 20), and re-probed.

### Transmission electron microscopy

PC-12 cells seeded at 1.5 × 10^5^ cells in a 60 mm plates (Nunc), transfected 16 h post seeding with pMT2 or MK-STYX, stimulated with 100 ng/μl NGF 24 h post-transfection. Control treatment was non-transfected PC-12 cells. Three days post-transfection cells were fixed with fixed with 2.5% glutaraldehyde in 0.1 M cacodylate, 10 mM KCl, 5 mM MgCl_2_, pH 7.2 for 12 h at room temperature. Cells were scraped, centrifuged at 3,000 RPM for 5 min to form a pellet, and post fixed with 2% OsO_4_ for 1 h. Samples were then infiltrated using 1:1 Spurrs and 100% ethanol at room temperature for 1 h. The samples were transferred to BEEM capsules, allowed to polymerize overnight at 60°C, sectioned, and stained with uranyl acetate and lead citrate, analyzed and imaged.

### Transient transfection and cell imaging

For immunofluorescence assays, PC-12 cells were plated at 2 × 10^4^ cells per well on type I collagen-coated coverslips (Neuvitro) in 6-well dishes (Nunc). Twelve to eighteen hour post-plating, cells were transiently transfected with 2 μg of pMT2 or FLAG-tagged MK-STYX expression plasmid DNA and 4 μl of Lipofectamine 2,000 per well, according to the manufacturer's protocol. The medium was replaced 5 h after transfection. Twenty-four hour post-transfection, cells were serum starved with DMEM supplemented with 0.1% FBS serum for 8–12 h and then stimulated with 100 ng/ml of NGF (Prospec) (medium was replaced every 48 h with treatment medium). Three days post-stimulation cells were prefixed in 2% formaldehyde for 2 min to preserve cell structure integrity, and then fixed in 3.7% formaldehyde 8–10 min. Cells were washed with PBS and permeabilized with 0.1% Triton-X-100 for 5 min. For experiments examining the effect of MK-STYX on microfilament and microtubule dynamics, cells were sequentially stained with rhodamine-conjugated phalloidin (1:200 dilution; Invitrogen) for 1.5 h, then probed with anti-mouse β-tubulin FITC (Sigma) for 1 h. To visualize whether MK-STYX-induced neurites have pre-synaptic and post-synaptic characteristics, cells were with the anti-MAP2, FITC conjugated (1:100 dilution; Bioss) dendrite marker for post-synapses, and the axonal marker anti-Tau1, Cy3 conjugated (1:150 dilution; Bioss) for pre-synapses. For the localization studies, cells were transfected with mCherry or pEGFP or mCherry-MK-STYX or GFP-MK-STYX. To visualize whether MK-STYX localized to post-synaptic regions, MAP2 was used as the marker and visualized with anti-MAP2, FITC conjugated (1:100 dilution; Bioss), and to visualize the localization of MK-STYX, cells were transfected with mCherry-MK-STYX. To visualize whether MK-STYX localized to pre-synaptic regions, Tau was used as the marker and visualized with anti-Tau conjugated to Cy3, and cells were transfected with GFP-MK-STYX to visualize the localization of MK-STYX. To determine whether MK-STYX-induced neurites altered the released of dopamine, a neurotransmitter released by PC-12 cells (Greene and Tischler, 1976), cells were stained with anti-dopamine antibody (1:200) (Abcam) and secondary anti-rabbit conjugated Cy3 antibody (1:250) (Cell Signaling). Post-staining, the coverslips were mounted to a slide using GelMount containing 4′, 6-diamidino-2′-phenylinodole dihydrochloride (DAPI, Sigma) (0.5 mg/ml).

Counting and image collection were performed on a Nikon ECLIPSE Ti inverted fluorescence microscope. NIS-Elements Basic Research software (version 3.10, Nikon) was used for image acquisition and primary image processing, and Adobe Photoshop, and Illustrator were used for secondary image processing.

### Dopamine assay

PC-12 cells were transfected with pEGFP or GFP-MK-STYX expression plasmids and lysed 3 days post NGF stimulation, and analyzed by immunoblotting. Cells were harvested in lysis buffer and quantified. LDN (Labor Diagnostika Nord) Dopamine Research ELISA™ was used to determine dopamine; the manufacturer protocol was followed.

### Fluorescence analysis

All fluorescence intensity measurements were made in ImageJ. To obtain the corrected total cell fluorescence (CTCF) the following formula was used: Integrated density-(Area of selected cell × mean fluorescence of background readings). To determine the ratios of MAP2 and Tau in the neurites, CTCF values were obtained for the whole cell (CTCF_*total*_) and the soma alone (CTCF_*soma*_). Fluorescence intensity of the neurites (CTCF_*neurites*_) was determined by subtracting the CTCF_soma_ from the CTCF_*total*_. The ratio of MAP2 or Tau present in the neurites was then determined by dividing the CTCF_neurites_ by CTCF_*total*_.

### Colocalization analysis

All colocalization analysis was performed in ImageJ using the Coloc2 plugin (https://imagej.net/Coloc_2), an automated system that evaluates the fluorescent intensities of every pixel in a given area. Quantification of colocalization was performed using Pearson's correlation coefficient. The Pearson's correlation coefficient reflects the degree of linear relationship between two variables; in this case, the fluorescence intensities of two fluorescently tagged proteins.

### Primary neuron cell culturing and electroporation

E18 Sprague Dawley rat hippocampal neurons were obtained from BrainBits and the BrainBits Complete Culturing Kit was followed. 10^6^ neurons were centrifuged at 1100 RPM, supernatant decanted, and resuspended in 100 μl intracellular (IBN) buffer solution (135 mM KCL 0.2 mM CaCl2, 2 mM MgCl2, 10 mM HEPES, 5 mM EGTA, ph 7.3), which was placed in a 2 mm gap electroporation cuvette (Bio-Rad). 2 μg cDNA (pMT2-MK-STYX or pEGFP and pMT2 and pEGFP, respectively was also added to the cuvette. The cuvette was placed inside the Gene Pulser® II Electroporation System, cells were voltage pulsed with 300 mV. Cells were immediately removed from the electroporation cuvette and a fraction of cells seeded on poly-D-lysine coverslips (for optimal neuronal attachment) and the remaining in poly-D-lysine treated 48-well assay ViewPlates^TM^ (Packard Instrument Company, Meriden, CT, USA). Cells were cultured in NbActive1 media (BrainBits), maintained at 37°C, 5% CO_2_ in an incubator and allowed to differentiate for 3–4 days.

Cells seeded on coverslips were analyzed 3 days post-electroporation. These cells were fixed 3.7% formaldehyde 8–10 min and analyzed by fluorescence microscopy as previously described to determine successful electroporation. Fifteen percent of cells were successfully electroporated. Live images were taken of cells placed in the 48-well plates 4 days post-electroporation. Cells were analyzed and scored for >5 primary neurite extensions.

### Neurite length analysis

Analysis of neurite length in primary neurons was performed in ImageJ using the NeuronJ plugin (https://imagej.net/NeuronJ) (Pool et al., 2008). The total neurite length in each field of view was determined by tracing of neurite paths, and normalizing outgrowth to the total number of cells present per field of view.

### Statistical analysis

**Figure 1:**
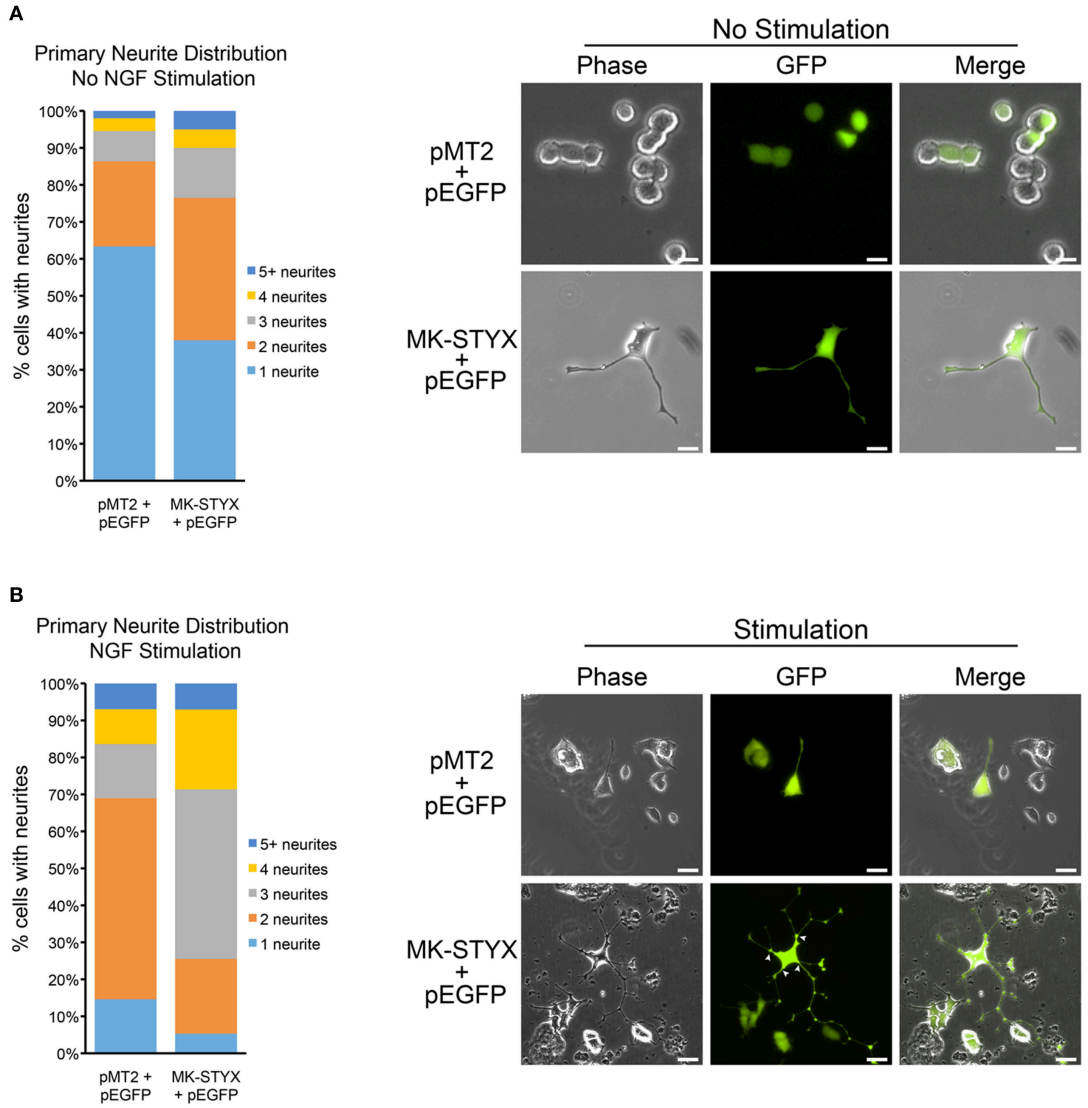
MK-STYX increases the number of primary neurites in PC-12 cells. **(A)** We scored the number of primary neurites (extensions from the cell body) ≥ 20 μm in PC-12 cells over-expressing GFP and MK-STYX or pMT2 control plasmid in the absence of NGF. Statistical analysis was performed (Chi-squared test: *p* < 0.005, 1 neurite); *p* < 0.01, 2, or 3 neurites). **(B)** PC-12 cells over-expressing GFP and MK-STYX or pMT2 control plasmid for 24 h were stimulated with 100 ng/ml NGF. 72 h post-stimulation cells were scored (*n* = 100) for primary neurites, and statistical analysis was performed (*p* < 0.005 for 1, 2, or 3 primary neurites). White arrows indicate primary neurites.

Statistical analysis was performed with GraphPad Prism software. Chi-squared test was used to compare the distribution of primary neurites in the presence of absence of NGF, to determine the statistical significance of differences between results with a significance level *p* < 0.05 (Figure 1). Paired *t*-test was use to compare MAP2 or Tau expression in cells stimulated with NGF in the presence or absence of MK-STYX (**Figure 4**); to compare the expression of dopamine in the absence of presence of MK-STYX (**Figure 7**); and to determine the significance of neurite outgrowth per neuron in the presence or absence of MK-STYX (**Figure 8**). The Pearson's Correlation Coefficient analysis was used to determine the colocalization of MK-STYX and MAP2; the One-way ANOVA (analysis of variance with Tukey's multiple comparison *post hoc* test was used to determine the statistical differences between these samples (**Figures 5, 6**). Data are presented as mean *r* ± SEM.

## Results

### MK-STYX increases the number of primary neurites in PC-12 cells

PC-12 cells do not differentiate and form neurites without a stimulus that sustains the ERK/MAPK (extracellular regulated kinase/mitogen-activated protein kinase) pathway (Qiu and Green, 1991; Qui and Green, 1992). NGF causes sustained activation of MAPK (Traverse et al., 1992; Keegan and Halegoua, 1993; Grewal et al., 1999; Daniele et al., 2010) and is well characterized as an inducer of neuronal differentiation of PC-12 cells (Traverse et al., 1992; Pang et al., 1995). Thus, we suspected that MK-STYX, which alone induces neurite-outgrowth in PC-12 cells (Flowers et al., 2014), might increase neurite differentiation. To analyze MK-STYX's effects on primary neurites (extensions originating from the cell body) in response to a neurotrophin, we stimulated cells co-expressing pEGFP and pMT2 or MK-STYX with NGF. To determine if MK-STYX alters the number of primary neurites, cells were imaged and categorized as having 1, 2, 3, 4, or ≥ 5 primary neurites. PC-12 cells were transfected with pEGFP and pMT2 or MK-STYX and observed for neurite outgrowth 3 days later. Cells were imaged and the extensions were measured from the cell body to the end as depicted and reported previously (Flowers et al., 2014). An extension ≥20 μm was considered to be a neurite-like outgrowth.

MK-STYX significantly increased the number of primary neurites extending from a cell (Chi-squared test: *p* < 0.05, 1 neurite; *p* < 0.01, 2 or 3 neurites) (Figure 1A). The majority of control cells stimulated with NGF formed 2 primary neurites. Most NGF-stimulated cells expressing MK-STYX formed 3 or 4 primary neurites (Chi-squared comparison test: *p* < 0.05), which is indicated with white arrows (Figure 1B). A pattern change, such as an increase in primary neurites is indicative of the morphological rearrangement of cytoskeletal proteins, such as microtubules and microfilaments (Dotti et al., 1988).

### MK-STYX alters PC-12 cell morphology and enhances growth cone formation

**Figure 2:**
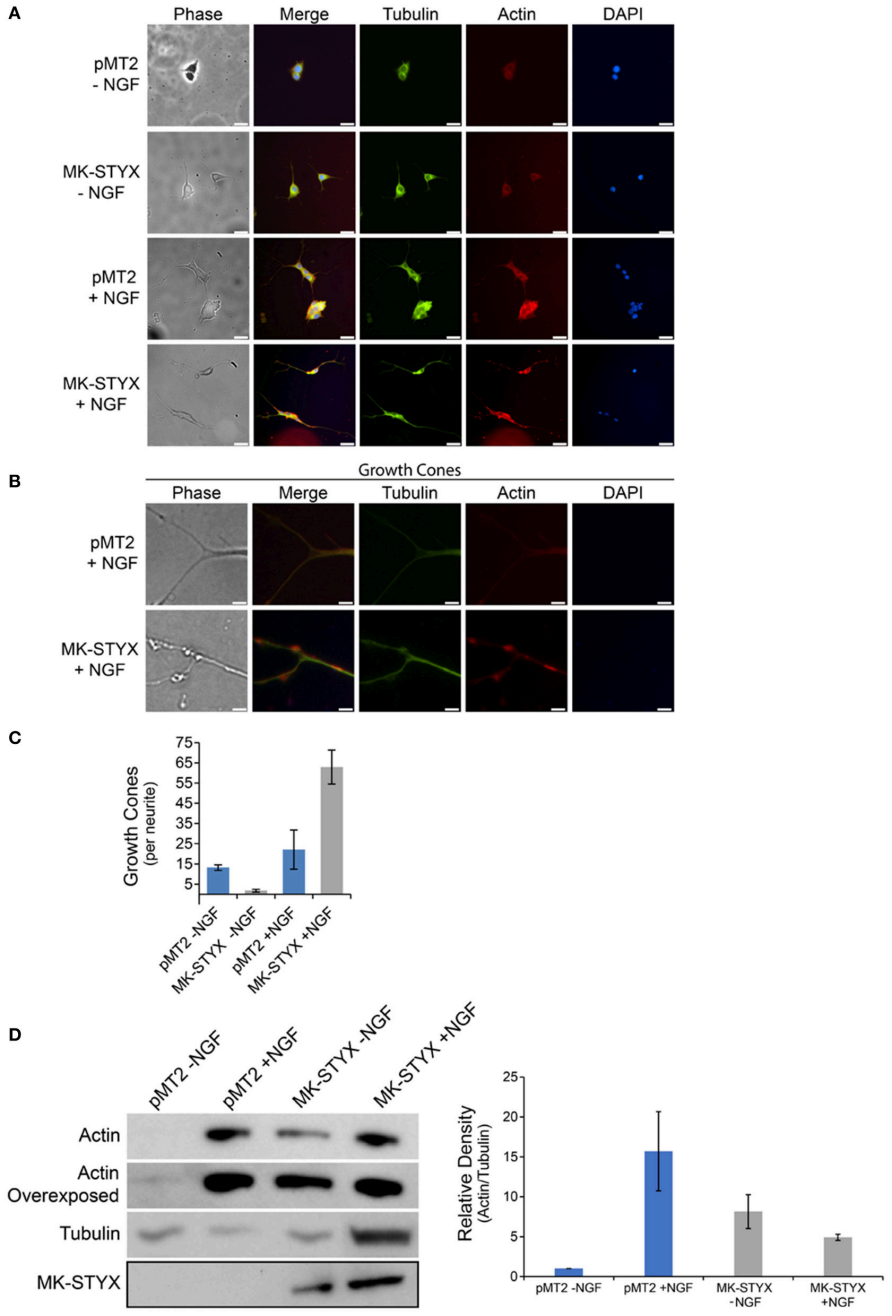
MK-STYX induces growth cones and actin protrusions in the neurite shaft. **(A)** Representative examples of the subcellular distribution of microtubules, which were detected with a tubulin antibody conjugated to FITC (anti-β-tubulin-FITC), and microfilaments, which were stained with rhodamine-conjugated phalloidin, in cells transfected with MK-STYX or the pMT2 (empty vector) in the presence or absence of NGF as indicated. **(B)** Higher magnification image of cells stimulated with NGF from **(A)** showing the abundance of the actin filaments at the distal ends, of neurites which are indicated as growth cones, and **(C)** We scored the number of growth cones per length of a neurite (extensions from the cell body) ≥ 20 μm in PC-12 cells over-expressing pMT2 (control plasmid) or MK-STYX in the absence or presence of NGF. Statistical analysis was performed (paired *t*-test: *p* < 0.005); the error bars are SEM. Five replicate experiments were performed by three different individuals. **(D)** PC-12 cells transfected with pMT2 or pMT2-FLAG-MK-STYX-FLAG and stimulated with NGF 2 days post-transfection. Three days post NGF stimulation cells were lysed and the proteins subjected to SDS-PAGE and immunoblotting. Anti-actin (C4) antibody showed that MK-STYX up-regulated actin relative to control in the absence of NGF. In contrast, MK-STYX prevented a further up-regulation of actin relative to the control in the presence of NGF. An overexposed actin blot is shown to demonstrate that actin is expressed in all conditions. The blot was stripped and probed with antibody anti-β-tubulin as a loading control. Three replicate experiments were performed. ImageJ was used to analyze blots; the graph is the average of the relative density of actin/tubulin for all three bots; the normalization is relative to pMT2 in the absence of NGF. Statistical analysis was performed (paired *t*-test; *p* < 0.05 for pMT2 –NGF compared to pMT2 + NGF or MK-STYX – NGF); the errors bars are SEM.

Changes in cell morphology are regulated by the organization of cytoskeletal networks, such as microtubules or actin microfilaments (Fuchs and Karakesisoglou, 2001). To determine whether MK-STYX alters these cytoskeletal elements, PC-12 cells expressing MK-STYX or pMT2 (control plasmid) were stained with an anti-β-tubulin antibody for microtubules or conjugated phalloidin for microfilaments. Microtubules were prominent in cells expressing MK-STYX and not stimulated by NGF compared to pMT2 control cells, where microfilament staining was stronger (Figure 2A). Furthermore, microfilaments were primarily confined to the cell body. In response to NGF, cells expressing MK-STYX showed increased f-actin staining, and both f-actin and microtubules were present throughout the length of the neurite, whereas in the control cells actin was mostly in the cell body and at the proximal end of a neurite (Figures 2A,B). Intriguingly, the actin and tubulin distribution in neurites induced by MK-STYX alone resembled neurites of control cells stimulated with NGF.

Cells expressing MK-STYX in response to NGF had several actin-rich protrusions along the neurites. Within the shafts, there were veil-like actin protrusions (Figure 2B), which are indicative of growth cones in neurites. Growth cones are specialized cytoskeletal regions that serve as guides for neurites to form proper synapses (Purves and Lichtman, 1985a; Kolodkin and Tessier-Lavigne, 2011). These growths cones were significantly more prevalent in NGF stimulated cells expressing MK-STYX, than in control cells (Figure 2C), perhaps providing a causative explanation for why more primary neurites are found in cells expressing MK-STYX. This also may explain the branching (where neurite extensions separate further) we observed previously (Flowers et al., 2014; Dahal and Hinton, 2017), and in the current study (Figures 1B, 2A,B). Growth cones may lead to splitting, where cytoskeletal protrusions split off from the end of a neurite or collateral branching where a growth cone arises along a neurite shaft (Gallo, 2011; Gibson and Ma, 2011); both neurite dynamics are seen in the presence of MK-STYX (Figures 1B, 2A,B). Actin expression was significantly increased (paired *t*-test; *p* < 0.05) in the presence of MK-STYX in the absence of NGF (Figure 2D), when actin expression is expected to be low, which was the case in the control (Figure 2D). Intriguingly, MK-STYX prevented further increase of actin expression in the presence of NGF, which elicits an increase in actin expression, as seen in the control NGF. Actin protrusions of the distal ends of neurites are considered to be an indication of dendritic development. Dendrite development was initially only considered as a result of alternate extension and branching (McAllister, 2000); however, dendrite development is much more dynamic. Moreover, the initial neurite extensions of primary neurons are important for both axon and dendrite guidance (Scott and Luo, 2001), and the dynamic process of extension is important for the formation of a synapse, where an axon reaches its target dendrite (Hume et al., 1983; Young and Poo, 1983).

### MK-STYX-induced neurites form synapse-like structures

**Figure 3:**
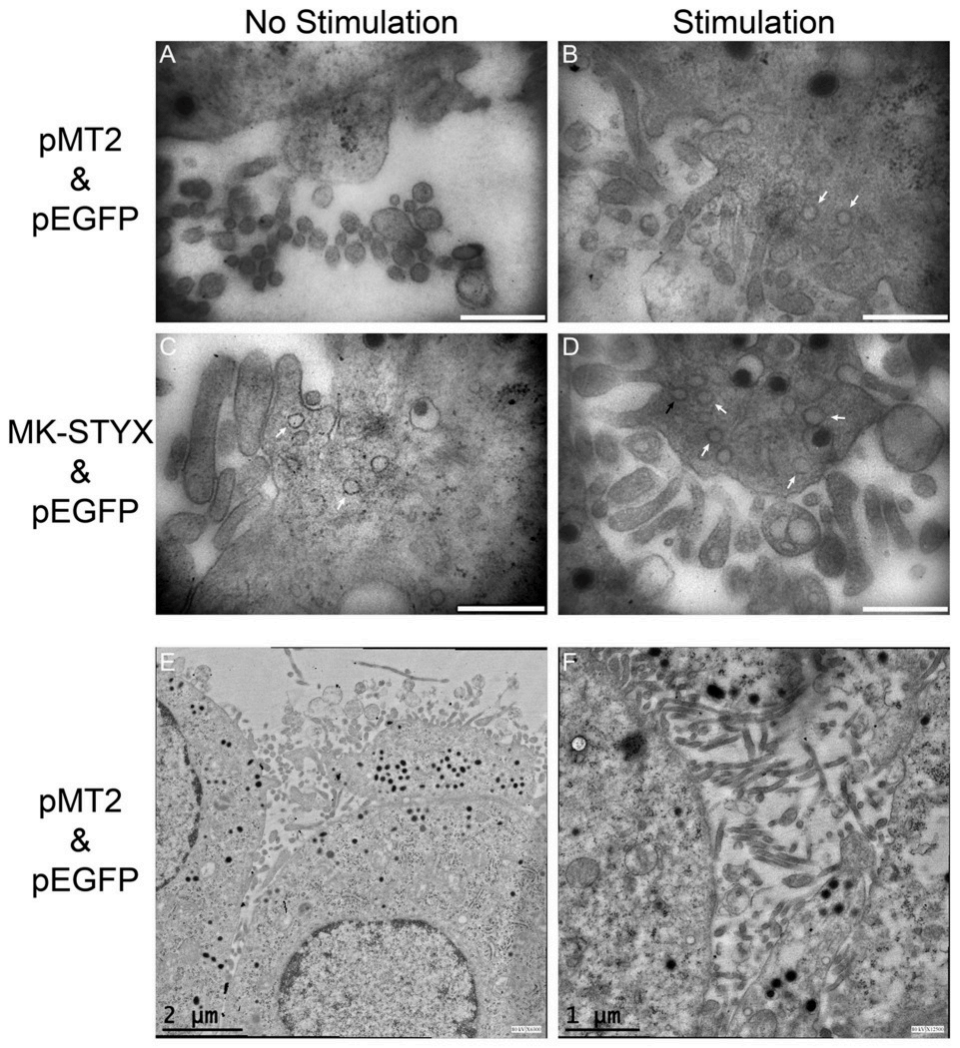
MK-STYX-induced neurites form synapse-like structures. Non-stimulated cells expressing **(A)** pMT2 and pEGFP expressing PC-12 cells did not form synapse-like structures. **(B)** By contrast, pMT2 and pEGFP expressing PC-12 cells stimulated with NGF formed synapses-like structures. Images taken from cross sections of connections clearly show many vesicles (white arrows) and clathrin-coated membrane invaginations (black arrows). Although NGF stimulated cells expressing pMT2 and pEGFP (control cells) formed synapse-like connections, many more such vesicles and clathrin-coated membrane invaginations formed in cells expressing both MK-STYX and pEGFP in the **(C)** absence or **(D)** presence of NGF. Scale bar is 500 nm for images **(A–D)**. **(E,F)** Higher magnification of PC-12 cells expressing pMT2 shows that connections were maintained during the processing for TEM.

We previously reported that MK-STYX enhances the effect of NGF by significantly inducing a much longer outgrowth (Flowers et al., 2014). These extensions appeared to connect, suggesting that synapse-like structures were forming. Therefore, we examined the effects of MK-STYX on PC-12 cells by transmission electron microscopy (TEM) to determine their synaptic status. Cells expressing pMT2 and pEGFP (control cells) did not form synapse-like structures even when two cells were next to each other (Figure 3A). However, synapse-like structures were observed in PC-12 cells stimulated with NGF (Figures 3B,D), and in non-stimulated cells overexpressing MK-STYX (Figure 3C). Cross sections of spine-like structures show that these cells have many vesicles and clathrin-coated membrane invaginations (black or white arrow heads, respectively) (Figures 3B–D), which is indicative of synapses in PC-12 cells (Jeon et al., 2010). Furthermore, PC-12 cells expressing only MK-STYX formed synapses similar to NGF-stimulated cells expressing pMT2 (Figure 3C). Images of PC-12 cells stimulated with NGF and overexpressing MK-STYX suggest a potential increase in the presence of more vesicles and extensions (Figure 3D). An extension of one cell also connected to another cell (Figure 3D; near 10 clock, beneath letter D), further illustrating that MK-STYX enhances the effects of NGF and may have a role in cytoskeleton organization. These same effects were seen by fluorescence microscopy (Figure 1B). It is noteworthy to mention that cells analyzed in panel 3D are the same cells imaged in 1B (cells were fixed and imaged before TEM preparation), validating that these connections between cells seen in the presence of MK-STYX form synapses. Furthermore, higher magnification of PC-12 cells expressing pMT2 shows that connections were maintained during the processing for TEM (Figures 3E,F). In particular, more intertwining of extensions are visible between cells with NGF (Figure 3F).

### MK-STYX-induced neurites have pre-synaptic and post-synaptic processes

**Figure 4:**
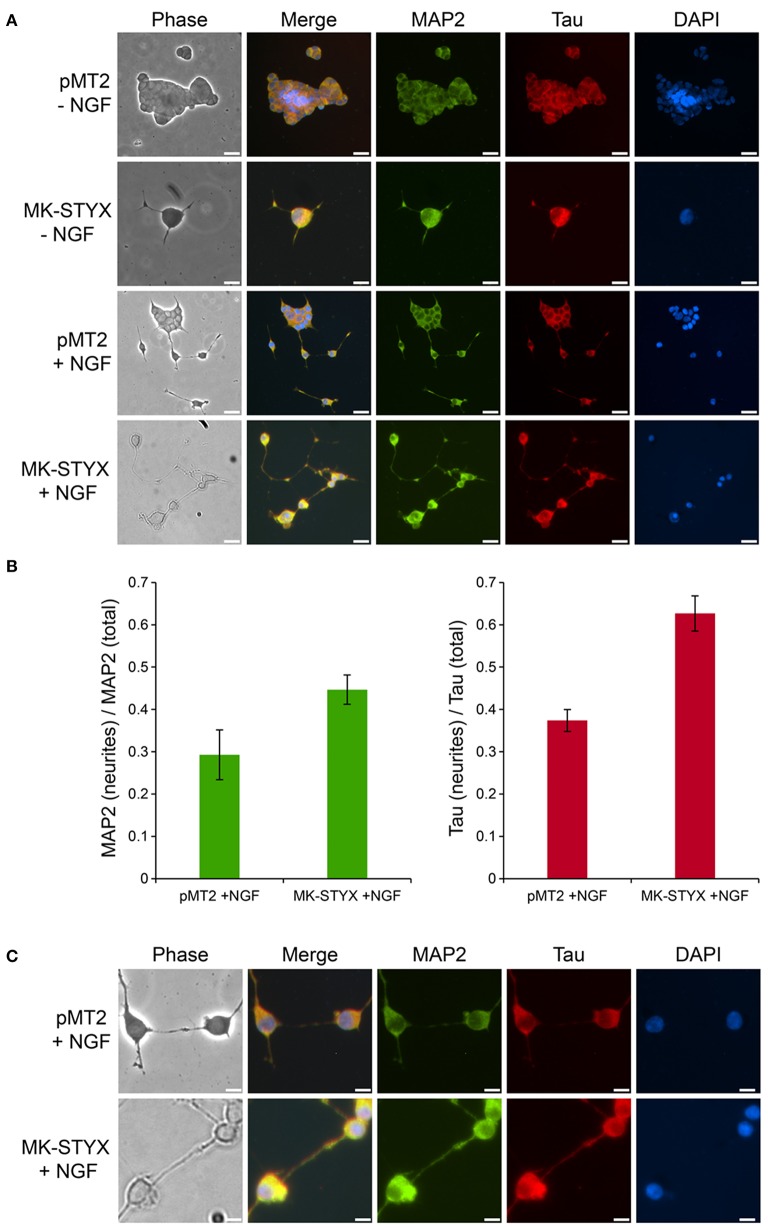
MK-STYX-induced neurites form pre-synaptic and post-synaptic connections. **(A)** PC-12 cells transfected with MK-STYX or control pMT2 plasmid vector were incubated in the presence or absence of NGF for 72 h post-transfection to allow neurite outgrowth, and stained with the dendritic MAP2 marker and the axonal Tau marker. Representative examples of MAP-2 immunoreactivity (green) show it in the cell body in non-stimulated cells, but also in major processes in stimulated cells or cells over-expressing MK-STYX. Tau (red) was localized to the cell body in non-stimulated cells, whereas it was more localized in neurites in NGF stimulated cells or MK-STYX expressing cells, demonstrating that stimulated control cells and MK-STYX expressing cells have pre-synaptic (axons-Tau) and post-synaptic (dendrites-MAP2) processes, and **(B)** fluorescence intensity measurements made in ImageJ. To obtain the corrected total cell fluorescence (CTCF) was used to determine the ratio of MAP2 or Tau in the neurites. MK-STYX significantly increased the localization of MAP2 (paired *t*-test; *p* < 0.05) or Tau (paired *t*-test *p* < 0.0005) in NGF stimulated cells; the errors bars are SEM. **(C)** In a higher magnification view of stimulated cells, the connections between the presynaptic and post-synaptic cells are visible and appear to be enhanced in cells expressing MK-STYX. Cells were fixed, stained with anti-MAP2 and Cy5-conjugated goat anti-rabbit antibodies, anti-Tau and Cy3-conjugated goat anti-mouse antibodies, and DAPI, and analyzed 72 h post-stimulation by fluorescence microscopy. Merged images show the localization of MAP2 (green: dendrite), Tau (red: axon), and DAPI-stained nuclei (blue). The phase image was not merged so that the cell morphology could clearly be seen. Scale bar, 50 μm. Five replicate experiments were performed by three different individuals.

To distinguish between axonal and dendritic-like processes in MK-STYX-induced neurites, the locations of axonal marker Tau and the dendritic marker MAP2 were determined. Both Tau and MAP2 were localized to the PC-12 cell body and processes (Figure 4), which is where these cytoskeletal proteins localize in PC-12 cells in response to NGF (Jeon et al., 2010). However, Tau was particularly localized in distal ends of neurites in cells expressing MK-STYX or stimulated by NGF (Figure 4), whereas in non-stimulated cells it was in the cell body. This suggests that these processes are axonal. MAP2 staining followed the same pattern (Figure 4A), suggesting that these processes are dendritic. Overall, Tau and MAP2 localization to the major processes were more prominent in NGF stimulated cells (Figure 4). Furthermore, the localization of MAP2 and Tau in the major processes was significantly more pronounced in the presence of MK-STYX (Figure 4B). MK-STYX expressing cells significantly increased the localization of MAP2 to neurites compared to control, with 44.66% of MAP2 expression localized to the neurites in the presence of MK-STYX compared to 29.28% in the control (Figure 4B) (paired *T*-test: *p* < 0.05). Tau localization to neurites also significantly increased in MK-STYX expressing cells compared to control, with 62.67% of Tau localized to the neurites in the presence of MK-STYX compared to 37.38% in the control (Figure 4B) (paired *t*-test; *p* < 0.0005). This pattern of distribution indicates that stimulated controls cells and MK-STYX expressing cells have pre-synaptic (axons-Tau) and post-synaptic (dendrites-MAP2) processes. Furthermore, Tau-containing processes formed a connection with MAP2-containing processes (Figure 4), which was observed in cells over expressing MK-STYX (Figures 4A,C).

### MK-STYX has a similar distribution pattern to the axonal marker Tau and the dendritic marker MAP2

**Figure 5:**
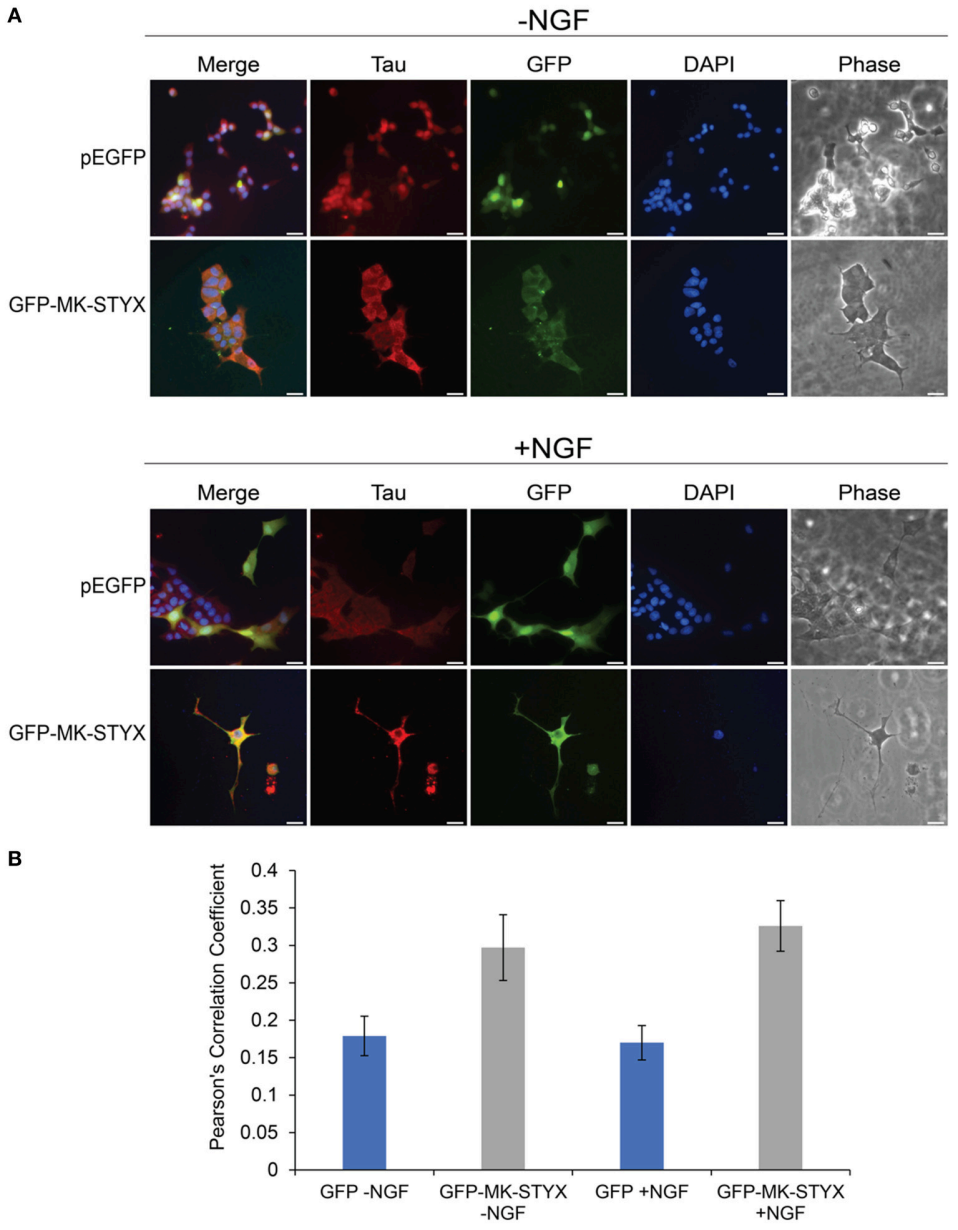
Co-localization of MK-STYX with the presynaptic marker protein, Tau. **(A)** Representative examples of the subcellular distribution of overexpressed MK-STYX with Tau in the absence (–NGF) or presence of NGF (+NGF). MK-STYX (green) and Tau (red) colocalized to the cell body in non-stimulated or NGF stimulated cells (even in the cells where MK-STYX induced neurite formation). Cells were fixed, stained with anti-Tau and Cy3-conjugated goat anti-rabbit antibodies, and DAPI, and analyzed 72 h post-stimulation by fluorescence microscopy. Merged images show the colocalization of endogenous Tau (red) relative to MK-STYX (green), and DAPI-stained nuclei (blue). The phase image was not merged so that the cell morphology could clearly be seen. Scale bar, 50 μm. **(B)** Colocalization analysis was performed in ImageJ using the Coloc2 plugin (https://imagej.net/Coloc_2). MK-STYX colocalization with Tau was determined by the Pearson's Correlation Coefficient. Statistical analysis was performed using ANOVA followed by Tukey's multiple comparison *post hoc* test (ANOVA *p* < 0.005). The error bars are SEM. Five replicate experiments were performed by two different individuals.

**Figure 6:**
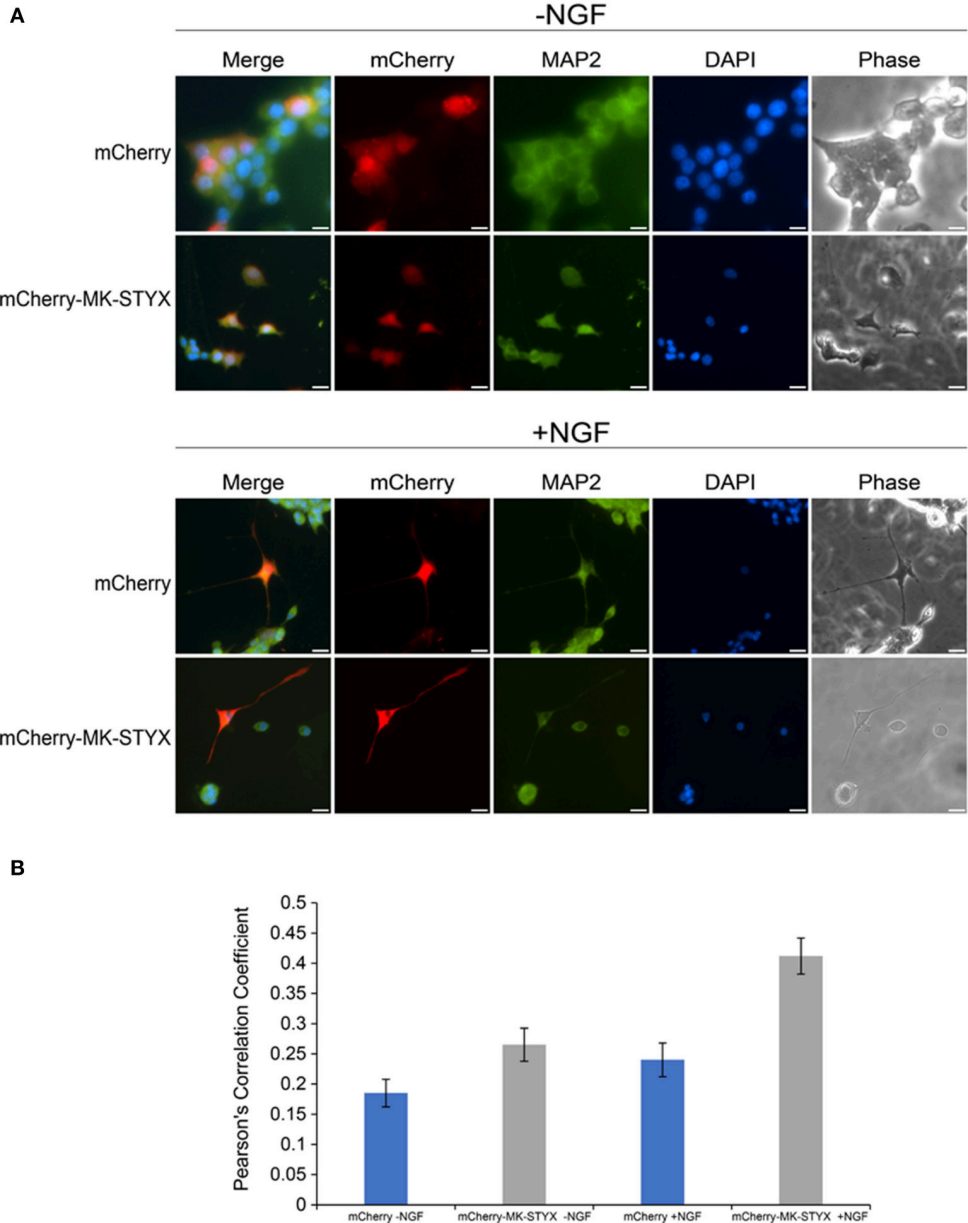
Localization of MK-STYX with the post-synpatic marker protein, MAP2. **(A)** Representative examples of the subcellular distribution of overexpressed MK-STYX with MAP2 in the absence (–NGF) or presence of NGF (+NGF). MK-STYX (red) colocalizes with the dendritic marker MAP2 (green) within in the cell body and the neurite length in NGF stimulated cells or non-stimulated cells transfected with mCherry or mCherry-MK-STYX. Cells were fixed, stained with anti-MAP2 and Cy5-conjugated goat anti-rabbit antibodies, and DAPI, and analyzed 72 h post-stimulation by fluorescence microscopy. Merged images show the localization of MAP2 (green: dendrite), MK-STYX (red) relative to each other, and DAPI-stained nuclei (blue). The phase image was not merged so that the cell morphology could clearly be seen. Scale bar, 50 μm. **(B)** Colocalization analysis was performed in ImageJ using the Coloc2 plugin (https://imagej.net/Coloc_2). MK-STYX colocalization with MAP2 was determined by the Pearson's Correlation Coefficient. Statistical analysis was performed using ANOVA followed by the Tukey's multiple comparison *post hoc* test (ANOVA) *p* < 0.005); the error bars are SEM. Five replicate experiments were performed by two different individuals.

Primary neurites were more numerous on PC-12 cells expressing MK-STYX (Figure 1), appeared to branch more (Flowers et al., 2014; Dahal and Hinton, 2017), and Figures 1B, 2B here), had actin rich processes along neurite shafts (Figure 2C), formed synapse-like structures (Figure 3) and had an asymmetric distribution of axonal (Tau) and dendritic (MAP2) markers (Figure 4B). Therefore, we performed experiments to assess whether MK-STYX colocalizes to the axonal or dendritic-like processes. In cells overexpressing MK-STYX, MK-STYX and Tau partially colocalized (Figure 5). This colocalization was significantly increased compared to control cells expressing GFP (Figure 5B) (Pearson's Correlation: One-way ANOVA with Tukey's multiple comparison *post hoc* test *p* < 0.05), indicating that the colocalization of Tau and MK-STYX is specific. MK-STYX colocalized to the cell body and the length of the neurites but Tau was not as prevalent in the full length of the “axonal” neurite of control cells in the presence of NGF (Figure 5). This pattern of colocalization to the cell body was also noticed with MK-STYX and the dendrite marker MAP2 (Figure 6). In NGF stimulated cells, overexpressed MK-STYX had similar distribution patterns with the MAP2, which is noted by the visibility of MK-STYX in some of the neurite branches (Figure 6). There was no significant change between the control mCherry overexpressing cells in the absence or presence of NGF, indicating that the colocalization of MK-STYX and MAP2 is specific. Moreover, the colocalization of MAP2 and MK-STYX in the presence of NGF is significant (Figure 6B) (Pearson's Correlation: ANOVA with Tukey's multiple comparison test *p* < 0.005), which was not the case for the colocalization of MK-STYX and Tau. The similar and significant localization pattern of MK-STYX to dendritic MAP2, compared with a lack of colocalization in the axonal and the branched areas, suggests that MK-STYX may be more involved in the development of dendritic processes.

### MK-STYX-induced neurites are dopaminergic

**Figure 7:**
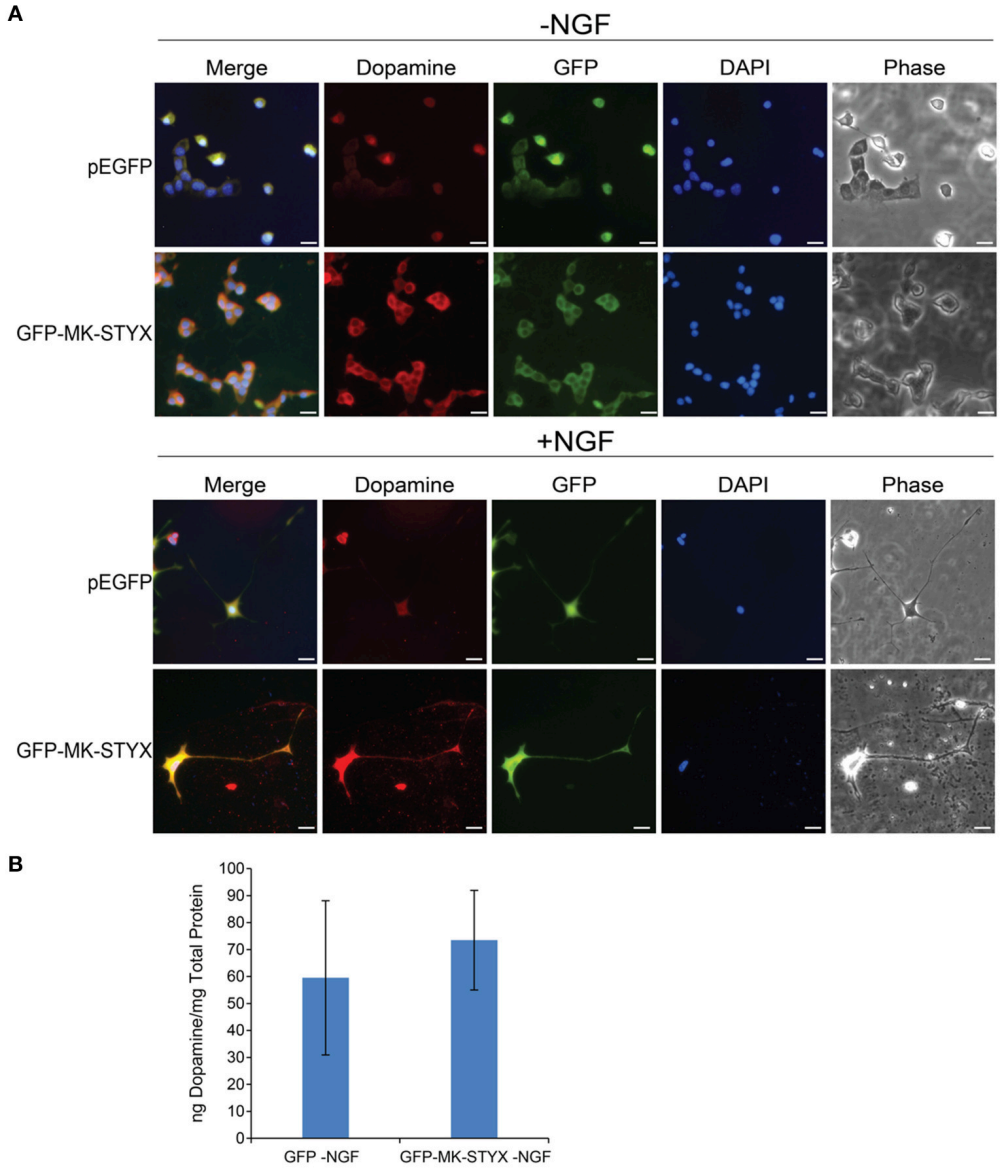
MK-STYX-induced PC-12 neurites are dopaminergic. **(A)** Representative examples of the subcellular distribution of dopamine in the presence or absence of MK-STYX (GFP-MK-STYX) in non-stimulated conditions (–NGF) or stimulated conditions (+NGF). Dopamine was expressed in all conditions. Cells were fixed, stained with anti-dopamine and Cy3-conjugated goat anti-rabbit antibodies, and DAPI, and analyzed 72 h post-stimulation by fluorescence microscopy. Merged images show the localization of dopamine (red), MK-STYX (green) relative to each other, and DAPI-stained nuclei (blue). The phase image was not merged so that the cell morphology could clearly be seen. Scale bar, 50 μm. **(B)** PC-12 cells were transfected with pEGFP or GFP-MK-STYX, lysed, and assayed for dopamine release. Dopamine release was determined with a Dopamine Research ELISA^TM^ (LDN) assay, and MK-STYX expressing cells released dopamine. Three replicate experiments were performed; the error bars are SEM.

The dopamine neurotransmitter has been well characterized in PC-12 cells (Greene and Tischler, 1976). PC-12 cells release dopamine and norepinephrine neurotransmitters for cellular communication (Greene and Tischler, 1976). Dopamine release in PC-12 cells is modulated by various factors, such as IA-2 (islet antigen 2) (Sai et al., 2013) and calcineurin (Kosiorek et al., 2014). Considering IA-2 and calcineurin are phosphatases and MK-STYX is a unique member of the phosphatase family, we wanted to determine whether MK-STYX-induced neurites were dopaminergic. Indeed, they were (Figure 7; GFP-MK-STYX in the absence of NGF). Dopamine was uniformly expressed throughout the cells for all conditions (Figure 7), which is expected. Furthermore, dopamine was released from control cells, as well as cells expressing MK-STYX (dopamine release was a small increase, but insignificant) (b)-indicating that MK-STYX-overexpressing cells maintain their dopaminergic nature.

### MK-STYX alters hippocampal primary neurons

**Figure 8:**
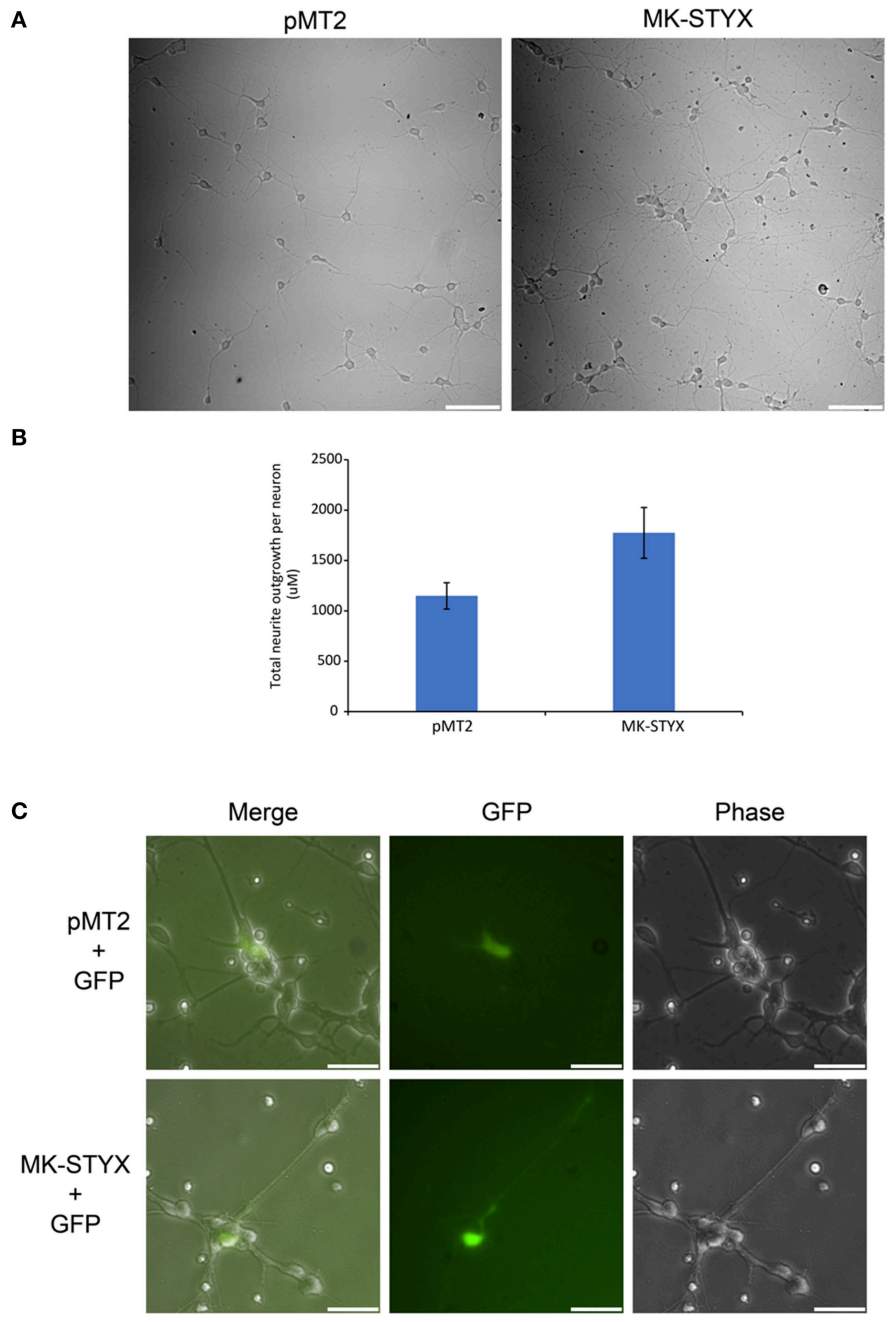
MK-STYX alters neuronal survival patterns. Representative examples of live cell images of hippocampal neurons in the absence or presence of MK-STYX. **(A)** Cells transfected with MK-STYX have more extensions coming from the soma, as well as more branching as compared to the control (pMT2). **(B)** Analysis of neurite length was performed in ImageJ using the NeuronJ plugin (https://imagej.net/NeuronJ). MK-STYX expressing neurons had a significant increase in the number of outgrowth neurites per neuron (paired *t*-test: *p* < 0.05); the error bars are SEM. **(C)** Live cell images of neurons co-expressing GFP and pMT2 or GFP and MK-STYX. GFP was localized to the extensions in the presence of MK-STYX, but only to the soma in the absence of MK-STYX. Three replicate experiments were performed.

**Figure 9:**
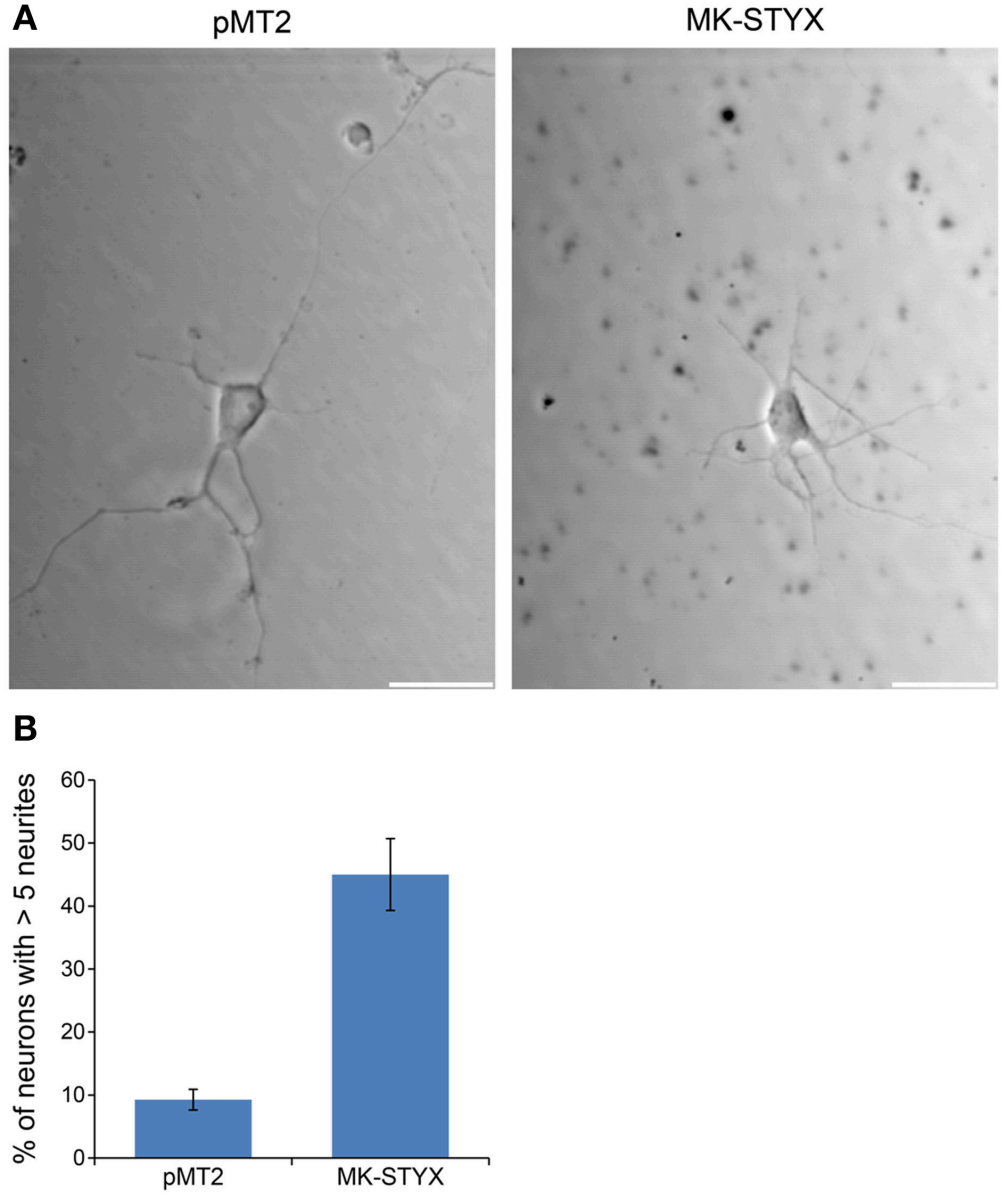
MK-STYX increases the number of primary neurites in neurons. Representative examples of live cell images of hippocampal neurons in the absence or presence of MK-STYX. **(A)** Cells transfected with MK-STYX show more debris and have a strikingly different morphology than control neurons, which had a distinguishable axon and dendrites **(B)** Neurons over-expressing GFP and pMT2 (control plasmid) or MK-STYX were scored seventy-2 h post-stimulation cells were scored (*n* = 50) for primary neurites, and statistical analysis was performed (paired *t*-test: *p* < 0.005); the error bars are SEM. Scale bar, 50 μm. Three replicate experiments were performed.

These studies performed in PC-12 cells provide significant and substantial data that the pseudophosphatase MK-STYX induces neuronal phenotypes. However, the question remained whether MK-STYX effects primary neurons and actually has a role in their development. Addressing this important question would simultaneously enhance our PC-12 findings, while providing insight into a role for MK-STYX in primary neurons. Therefore, we investigated whether MK-STYX altered the morphology of primary neurons. MK-STYX expressing primary neurons were strikingly different from the control neurons (Figure 8). Although cells were seeded at the same concentration, control cells differentiated into separate neurons forming distinguishable neurite connections to other neurons (Figure 8A). However, cells expressing MK-STYX grew in clumps with a complicated web-like connection of neurons (Figure 8A); there were significantly more neurites per neurons in cells expressing MK-STYX (Figure 8B); MK-STYX expressing cells also had more neurons per field (data not shown). Similar to MK-STYX expression in PC-12 cells, MK-STYX, and pEGFP expression was throughout the length of a primary neurite, whereas the pMT2 and pEGFP were mostly found in the cell body (Figure 8C). An axon (longest neurite) could clearly be distinguished in the control neurons (Figures 8, 9A). In contrast, an axon could not be distinguished in neurons expressing MK-STYX (Figures 8, 9A). Neurites from MK-STYX expressing neurons overlapped and intertwined with each other, a phenomenon that normally does not happen because neurons express adhesion molecules that participate in hemophilic binding and self- avoidance regulation mechanism to prevent extensions from the same neuron intertwining (Zipursky and Grueber, 2013). Furthermore, MK-STYX expressing neurons developed significantly more than the normal five neurites expected of a neuron (Figure 9B) (Dotti et al., 1988; Baas et al., 1989). More cells per area and a great deal of cellular debris were noticeable in the presence of MK-STYX, compared to control (Figures 8A, 9). MK-STYX's role in cellular survival and as a master regulator of apoptosis (Niemi et al., 2011, 2014) supports the observation of more cellular debris observed in the presence of MK-STYX.

## Discussion

In the present study, we analyzed these MK-STYX-induced neurite-like outgrowths further in PC-12 cells and in rat primary neurons. Considering that PC-12 cells are not primary neurons, it was important to determine whether these MK-STYX-induced “neurites” develop neuronal characteristics, such as formation of synapses and portrayal of presynaptic and post-synaptic properties. We show that MK-STYX increases the number of primary neurites extending from a cell body. Furthermore, we show that MK-STYX-induced neurites significantly increased actin growth cones throughout their shafts. Electron microscopy demonstrated that MK-STYX-induced neurites formed connections that are synaptic, which is represented by the secretory vesicles, and vesicle fusion with the plasma membrane near the contact area. Furthermore, PC-12 cells expressing MK-STYX formed synapses similar to NGF control cells only expressing pMT2. Staining with the axonal and dendritic makers, Tau and MAP2, respectively, show that MK-STYX-induced neurites have presynaptic and post-synaptic properties, and may form presynaptic and post-synaptic connections. Taken together, these findings provide evidence that MK-STYX-induced outgrowths fit the criteria for neurites. Over-expressed MK-STYX appears to localize abundantly at the post-synaptic process within these MK-STYX-induced neurites, with a similar distribution pattern to MAP2, and are dopaminergic.

Lastly, we present novel and significant data that MK-STYX alters the morphology of hippocampal neurons. Overexpression of MK-STYX caused more connections between primary neurons, a significant increase in primary neurites, as well as more cellular debris. MK-STYX is an apoptotic regulator (Niemi et al., 2011, 2014); therefore, this cellular debris may be an important factor. The data obtained in primary neurons enhances our PC-12 findings, while solidifying and edifying the foundation for understanding pseudophosphatase MK-STYX in neuronal development.

Neuritogenesis (neurite formation) is the first event in neuronal differentiation (Govek et al., 2005). It is achieved through the ability of cells to change their shape, which is dependent on the assembly and dynamics of the cytoskeleton. There are two basic requirements for neurite formation: a dynamic actin network and bundling of microtubule arrays (Flynn, 2013). Here, we show that MK-STYX altered the dynamics of both microfilaments and microtubules in PC-12 cells. MK-STYX alone had a more robust effect on microtubules, but in the presence of NGF it had a more robust effect on microfilaments. Furthermore, these actin rich protrusions were organized into visible growth cones. Growth cones are composed of finger-like filopodia (bundled F-actin fibers) and veil-like lamellipodia (cross-linked network of microfilaments), which are at the tips of developing neurites, and were first reported by Ramón y Cajal in 1890 (as reviewed in Purves and Lichtman, 1985b). Growth cones serve as guidance for neurites to make the proper synapses with their partners (Kolodkin and Tessier-Lavigne, 2011; Santiago-Medina et al., 2015).

This abundance of growth cones in cells over-expressing MK-STYX may explain why these cells also form multiple primary neurites. We noticed that PC-12 cells over-expressing MK-STYX had multiple primary neurites, suggesting that MK-STYX may induce a distinguishable pattern of neuritogenesis in these cells. This is an important finding because neurites later become axons and dendrites, ultimately resulting in neuronal function (Sanes et al., 2012). Therefore, MK-STYX potentially may induce more dendrites to form neuronal connections; we hypothesize that more dendrites are induced because only one neurite specifically becomes an axon, except in the case of supernumerary axons (Tremblay et al., 2010; Reiterer et al., 2017). It is important to remember that growth cones are axonal and become pre-synaptic upon contact with a post-synaptic partner (Purves and Lichtman, 1985b). MK-STYX's effects on microfilament dynamics are also visible in the neurite distal ends in response to NGF. However, these are smaller actin protrusions, which may be indicative of branching (Flowers et al., 2014). Intriguingly, these small actin protrusions may also be indicative of post-synaptic dendritic spines. The increase of actin expression in the presence of MK-STYX validates that MK-STYX has a role in microfilament dynamics. Moreover, MK-STYX prevents a drastic increase in actin expression when expected with NGF stimulation. This suggests that MK-STYX may be a modulator of actin expression, further complimenting and strengthening our previous report that RhoA activation is decreased in the presence of MK-STYX (Flowers et al., 2014).

Taken together with the fact that branching is the most notable feature of dendrites (Hausser et al., 2000), and that MK-STYX significantly colocalizes more with the dendritic marker and causes spine neck elongation in primary rat neurons (unpublished data), it may have a role in dendritic spine formation, which is important for neuronal communication (Hausser et al., 2000). Synapses are where neuronal cells communicate with each other (Purves and Lichtman, 1985b). PC-12 cell neurites induced by factors, such as NGF, bFGF, and cAMP form synapse-like structures (Jeon et al., 2010). Our electron microscope study demonstrates that MK-STYX –induced neurites form synapse-like connections. Furthermore, our studies with the axonal and dendritic markers indicate that MK-STYX-induced neurites also have presynaptic and post-synaptic asymmetric distribution.

Moreover, MK-STYX's effects on the hippocampal primary neurons were dramatic and significant. The induction of more than the normal five neurites in a primary neuron resembles the induction of neurites in PC-12 cells. However, this phenomenon in primary neurons provides more insight. These neurites overlap and intertwine with each other, and no distinguishable axon could be determined, indicating that overexpressing MK-STYX may disrupt mechanisms for normal axonal formation. Intertwining and overlapping raises the question of whether MK-STYX affects self-recognition proteins along the neurite extension, which prevent neurites from a single neuron communicating with its own neurites (Zipursky and Grueber, 2013). The impact of MK-STYX on primary neurons confirms and validates our previous and current studies in PC-12 cells that MK-STYX may assist in rearranging the cytoskeletal components. Furthermore, MK-STYX's presence in primary neurite extensions may be indicative of components that MK-STYX interacts with to modulate cellular mechanisms.

In conclusion, we illustrate that outgrowths in PC-12 cells induced by MK-STYX develop a neuronal phenotype, and thus fit the criteria for bona fide neurites. Our studies also show that MK-STYX has a dramatic effect on microfilaments. The prevalence of growth cones in the presence of MK-STYX and the increase of actin expression in the absence of NGF is consistent with our previous report that MK-STYX inhibits RhoA activation, and decreases cofilin phosphorylation in the absence of NGF (Flowers et al., 2014), which is required for the induction of neurites (Zhang et al., 2006). The change in neurite pattern of hippocampal neurons in the presence of MK-STYX strengthens and creates an urgency to understand MK-STYX as a regulator in RhoA signaling. The molecular mechanism by which MK-STYX inhibits Rho activation remains elusive. However, our findings reported here strengthen our working model that MK-STYX acts through the RhoA signaling pathway (Flowers et al., 2014). For example, many of the processes MK-STYX elicits in this study, increasing primary neurites, branching, altering microfilament dynamics, formation of presynaptic and post-synaptic processes, are regulated by RhoA signaling (Govek et al., 2005). Given that the MK-STYX-induced neurites in PC-12 cells have neuronal properties, PC-12 cells can serve as an ideal model to understand the molecular mechanism of MK-STYX in neuronal differentiation and to study in correlation with primary neurons, which may provide insight into how pseudophosphatases exert their important roles in the pathology of neurodegenerative diseases. Future work is needed to examine the effects that silencing MK-STYX in primary neurons will elicit and to identify the mechanism by which MK-STYX regulates neurite formation. Recently, there have been a number of correlations pointing toward a key role of MK-STYX in neuronal development. MK-STYX is highly expressed in human hippocampus (Boycott and Wassle, 1991; Hubbard et al., 2012), and a missense mutation of *MK-STYX* results in intellectual disability and epilepsy (Boycott and Wassle, 1991), highlighting the timeliness of this study and the emergence of MK-STYX as an important candidate to understand the etiology of various neurodegenerative diseases.

## Author contributions

SH is responsible for developing the questions for the study, training and supervising the five undergraduates who participated in the study. She also performed the experiments for the TEM (Figure 3) and the initial experiments for the primary neurons (Figures 8, 9). She also analyzed data, wrote and edited the manuscript. DB performed the initial experiments for Figures 1–6, and performed the Chi-square analysis. He also assisted with editing the manuscript. AD repeated DB's initial studies to obtain better quality pictures for a resubmission. She also completed scoring and statistical analyses for Figure 2, as well as the statistical analysis for Figure 9. She performed the studies for the dopamine studies for Figure 7, and helped performed experiments (two out of three) for Figure 8. AM developed the protocol to successfully immunostain PC12 cells and primary neurons. BF provided GraphPad software and performed the initial study for Figure 1A and completely the initial statistical analysis. CS assisted with scoring growth cones for Figure 2. BS performed the dopamine assay for Figure 7B. AG prepped, analyzed, and imaged data for TEM. WA provided insight into the phalloidin studies and provided his expertise and facilities for TEM.

### Conflict of interest statement

The authors declare that the research was conducted in the absence of any commercial or financial relationships that could be construed as a potential conflict of interest.
